# Dissipation of field-aligned currents in the topside ionosphere

**DOI:** 10.1038/s41598-022-21503-x

**Published:** 2022-10-13

**Authors:** Fabio Giannattasio, Giuseppe Consolini, Igino Coco, Paola De Michelis, Michael Pezzopane, Alessio Pignalberi, Roberta Tozzi

**Affiliations:** 1 Istituto Nazionale di Geofisica e Vulcanologia, Via di Vigna Murata, 605, 00143 Rome, Italy; 2 INAF-Istituto di Astrofisica e Planetologia Spaziali, Via del Fosso del Cavaliere, 100, 00133 Rome, Italy

**Keywords:** Space physics, Atmospheric science

## Abstract

Field-aligned currents (FACs) are electric currents parallel to the geomagnetic field and connecting the Earth’s magnetosphere to the high-latitude ionosphere. Part of the energy injected into the ionosphere by FACs is converted into kinetic energy of the surrounding plasma. Such a current dissipation is poorly investigated, mainly due to the high electrical conductivity and the small electric field strength expected in direction parallel to the geomagnetic field. However, previous results in literature have shown that parallel electric field is not null (and may be locally not negligible), and that parallel electrical conductivity is high but finite. Thus, dissipation of FACs may occur. In this work, for the first time, we show maps of power density dissipation features associated with FACs in the topside ionosphere of the Northern hemisphere. To this aim, we use a 6-year time series of data at one second cadence acquired by the European Space Agency’s “Swarm A” satellite flying at an altitude of about 460 km. In particular, we use data from the Langmuir probe together with the FAC product provided by the Swarm team. The results obtained point out that dissipation of FACs, even if small when compared to that associated with horizontal currents flowing about 350 km lower, is not null and shows evident features co-located with electron temperature at the same altitude. In particular, power density dissipation features are enhanced mainly in the ionospheric regions where intense energy injection from the magnetosphere occurs. In addition, these features depend on geomagnetic activity, which quantifies the response of the Earth’s environment to energetic forcing from magnetized plasma of solar origin.

## Introduction

In the study of phenomena relevant to Space Weather, a crucial aspect is the characterization of the amount of energy deposited by field-aligned currents (FACs) in the high-latitude ionosphere, which triggers several processes inherent to the magnetosphere-ionosphere coupling. Indeed, it is well known that FACs carry energy and momentum into (and out of) the ionosphere that can be partially converted into mechanical energy and dissipated via heating^1^. Such a dissipation can significantly affect temperature, density and composition of the upper ionosphere resulting, for instance, in a change of satellite drag^2^.

The conservation of energy in a plasma volume is expressed by the Poynting’s theorem, which gives the relation between the energy density stored into an electromagnetic field, *W*, the energy flux quantified by the Poynting vector, ${\mathbf {S}}$, and the work done by the fields on a charge distribution, $\mathbf {J\cdot E}$, i.e. the energy dissipation. In differential form, the original Poynting’s theorem reads

1 $$\begin{aligned} \frac{\partial W}{\partial t} = -\mathbf {\nabla \cdot S}-\mathbf {J\cdot E}, \end{aligned}$$

where $W=(B^2/2\mu _0+\varepsilon _0E^2/2)$, $\mu _0$ and $\varepsilon _0$ are the vacuum permeability and permittivity, respectively, ${\mathbf {B}}$ and ${\mathbf {E}}$ are the magnetic and electric fields, respectively, ${\mathbf {S}}=(\mathbf {E\times B})/\mu _0$, and ${\mathbf {J}}$ is the current density. The second term in the right-hand side of Eq. (1), $\mathbf {J\cdot E}$, represents the rate of conversion of electromagnetic energy into mechanical energy. In a single-fluid magnetohydrodynamic (MHD) description of the ionospheric plasma this term assumes, in general, a complex definition that originates from the generalized Ohm’s law (GOL) and incorporates the contribution from electric and magnetic fields, pressure gradients, viscous stresses and momentum transfer via Coulomb collisions from electrons to ions and vice versa^3^. Under the reasonable assumptions of plasma quasi-neutrality, electron mass negligible with respect to the ion mass, and neglecting higher order terms, GOL can be simplified and reads^3^

2 $$\begin{aligned} {\mathbf {J}}=\sigma (\mathbf {E+u\times B}), \end{aligned}$$

where $\sigma$ is the electrical conductivity and ${\mathbf {u}}$ is the plasma bulk velocity. In direction parallel and perpendicular to ${\mathbf {B}}$, Eq. (2) provides, respectively

3 $$\begin{aligned} J_{||}=\sigma _{||}E_{||}, \end{aligned}$$

4 $$\begin{aligned} J_i=\sigma _{i,j}(E^j+\varepsilon ^{jkl}u_kB_l). \end{aligned}$$

In Eq. (4) we adopted the index notation, $\varepsilon ^{jkl}$ being the Levi-Civita tensor^4^, which selects only the components of ${\mathbf {u}}$ perpendicular to ${\mathbf {B}}$. It is worth underlining that the quantity in parenthesis in the right-hand side of Eq. (4) is the electric field in the plasma frame of reference. In direction parallel to the magnetic field, the quantity $\mathbf {J\cdot E}$ can be expressed by $\sigma _{||}E_{||}^2$, or equivalently, by the ratio $J_{||}^2/\sigma _{||}$, where $J_{||}$ coincides with FAC density strength and $\sigma _{||}$ is parallel electrical conductivity. In the F layer, even if small, a non-zero $E_{||}$ can be measured (see, e.g., Israelevich and Ofman^5^ and references therein), which provides a non-null $J_{||}^2/\sigma _{||}$. On the other hand, in direction perpendicular to the magnetic field $J_\perp$ is negligible at Swarm altitudes. In fact, Hall and Pedersen current densities quickly decrease with altitude, and at about 200 km are already 5 times lower than at their peak at about 100–120 km. In addition, during quiet conditions the maximum current density may be reduced by as much as a factor of 10 (see, e.g., Kamide and Brekke^6^, Casey^7^). The lower-order perpendicular diamagnetic current density, $J_d$, which is proportional to the pressure gradient, also provides a small contribution to $\mathbf {J\cdot E}$, namely $J_d^2/\sigma _\perp$, with respect to $J_{||}^2/\sigma _{||}$. By considering that at Swarm altitudes: (a) FAC density is of the order $10^{-6}$ A m$^{-2}$ (see section “Results and discussion”), (b) $\sigma _{||}$ is of the order of 10 S/m (see Giannattasio et al.^8^), (c) $J_d$ is of the order of 10$^{-10}$ A m$^{-2}$ (see Lovati et al.^9^), and (d) $\sigma _\perp$ can be approximated with the Pedersen conductivity, which is of the order of $10^{-6}$ S/m (see Maeda^10^), we obtain that $J_{||}^2/\sigma _{||}$ is about 10 times higher than $J_d^2/\sigma _\perp$. For this reason, the main contribution to $\mathbf {J\cdot E}$ at Swarm altitudes is given by $J_{||}^2/\sigma _{||}$.

To date, several studies have investigated the contribution of current dissipation to the energy budget of the ionosphere by using satellite data. For example, Heelis and Coley^11^ discussed the relation between dissipation rate via Joule heating and ion temperature at altitudes between 350 and 550 km. In particular, measurements of ion velocity from the Dynamics Explorer 2 (DE2) satellite, together with an estimation of the height-integrated Pedersen conductivity, allowed the authors to infer both the local and integrated dissipation rate. Kelley et al.^12^ computed the Poynting flux and estimated the dissipation via Joule heating from electric field measurements acquired by the HILAT satellite. The application of Poynting’s theorem allowed them to infer constraints on energy exchanges in the upper atmosphere. Chun et al.^13^ developed a method for estimating dissipation via Joule heating based on data from worldwide magnetometers and electrical conductivity obtained from particle flux measurements made on-board the NOAA TIROS and Defense Meteorological Satellite Program satellites. They found a proxy relationship between dissipation and the Polar Cap index^14^. Lühr et al.^15^ analyzed CHAMP data and found that small-scale FAC filaments with 1-km size and $J_{||}$ hundreds of $\upmu$A/m$^2$ can play a relevant role in dissipation via Joule heating. Weimer^16^ used electric and magnetic field measurements from DE2 to develop an empirical model of the high-latitude electric potential and FACs through a hybrid technique based on spherical harmonic functions at the pole and multiple Fourier series functions at low latitudes. He applied the electrodynamic model to calculate the Poynting’s flux and the dissipation rate via Joule heating without any conductivity model. Robinson and Zanetti^17^ calculated the auroral energy flux and the dissipation rate via Joule heating in the high-latitude ionosphere for 27 geomagnetic active days by using two-dimensional maps of FACs obtained by analyzing AMPERE data. They found that the energy due to dissipation increases more rapidly with geomagnetic activity than that due to particle precipitation. They also found that impulsive dissipation events correlate well with Sym-H index during the recovery phase of a geomagnetic storm. Pakhotin et al.^18^ used Swarm data to compute the electric field and point out a preference for electromagnetic energy input at 450 km altitude into the Northern hemisphere over the Southern hemisphere.

Here, for the first time we provide an estimate of the power density dissipated by FACs in the F layer of the Northern hemisphere, namely $\mathbf {J\cdot E}\simeq J_{||}^2/\sigma _{||}$, by using in situ observations only. Our purpose is to answer the questions: What is the amount of this physical quantity in the topside ionosphere? Is it negligible? Does it exhibit a noisy and salt-and-pepper structure due to fluctuations alternating in sign around the null value? Is it instead characterized by coherent features? If so, are they consistent with other observations at the same altitudes? To this aim, we used recently published maps of parallel electrical conductivity derived from electron density and temperature data (instead of models)^8,19^ together with time series of FAC density with the same cadence obtained by Swarm A measurements. The dependence of dissipation features on geomagnetic activity is also investigated.

## Data and methods

### The data set

We used electron density ($n_e$) and temperature ($T_e$) Level 1b data at 1 Hz acquired by the Langmuir Probes (LPs), part of the Electric Field Instrument (EFI) on board the Swarm A satellite^20,21^ of the European Space Agency from 1 April 2014 to 31 March 2020, when the satellite flew along a nearly circular and polar orbit with an inclination of $\sim$ 87.4$^\circ$ at an average altitude of $\sim$ 460 km. In situ $n_e$ and $T_e$ measurements, in Earth-centered geographic coordinates, were filtered out on the basis of the quality flags provided by the mission team. Specifically, we selected $n_e$ and $T_e$ data flagged with $Flag\_LP$=1 and $Flags\_T_e$ or $Flags\_N_e$ parameters equal either to 10 or 20. We also used Swarm A data from the Level 2 FAC-single product^22^, which provided the field-aligned current density strength, $J_{||}$, with the cadence of 1 Hz.

As we are interested in studying processes that crucially involve the geomagnetic field, we used the magnetic and non-orthogonal Quasi Dipole (QD) system of coordinates^23^ and accounted for the position of the Sun by using Magnetic Local Time (MLT) instead of UTC time. The transformation to QD coordinates consists of two steps: firstly, a transformation from geocentric latitude, longitude, and satellite altitude to geodetic latitude, longitude, and altitude; secondly, the transformation from geodetic to QD coordinates^24^.

### Power density dissipation of field-aligned currents

When electrons (the charge carriers^25,26^) embedded in FACs cross the ionospheric medium at Swarm altitudes, their momentum undergoes variations due to the interaction with electric and magnetic fields, pressure gradients, stresses and collisions mainly with the ions therein. In particular, collisional friction is responsible for the scattering of electrons along random directions and their following thermal motions. The momentum variation transfers energy to the surrounding plasma. As argued above, the power dissipated per unit volume along the direction parallel to the geomagnetic field is $J_{||}^2/\sigma _{||}$. $J_{||}$ is directly provided as a Swarm product, while $\sigma _{||}$ can be computed at Swarm altitudes under reasonable hypotheses^8,19^. In fact, by assuming that (i) ion species consist mostly of O$^+$ with the corresponding density similar to that of electrons^27^; (ii) the contribution of conductivity perpendicular to the main field is negligible with respect to parallel conductivity^28^; (iii) the electron mass is negligible with respect to ion mass; (iv) electron-ion Coulomb collisions dominate over elastic collisions with neutrals^29^ , the following expression holds for the parallel electrical conductivity^29^:

5 $$\begin{aligned} \sigma _{||}=\frac{e^2T_e^{3/2}}{\left[ 34+4.18\mathrm {ln}\left( \frac{T_e^3}{n_e}\right) \right] m_e}, \end{aligned}$$

where *e* is electron charge, $T_e$ electron temperature, $n_e$ electron density, and $m_e$ electron mass. As we can see, $\sigma _{||}$ depends only on $n_e$ and $T_e$, which are both measured by the Swarm LPs. Thus, Eq. (5) can be reliably used to compute $\sigma _{||}$ at Swarm altitudes, as in Giannattasio et al.^8^. The resulting conductivity can be used to obtain the power density dissipated by FACs at each satellite position. The time series of $J_{||}$, $J_{||}^2/\sigma _{||}$ and $T_e$ have been mapped as grids binned at 1$^\circ \times$1$^\circ$ in QD latitude vs MLT coordinates, where 1$^\circ$ in longitude corresponds to 4 min in MLT. We considered only QD latitudes above 50$^\circ$. The set of values collected within each bin, $\{w_i\}$, has been filtered out by using a simple median filter in order to remove outliers. Specifically, the filter works into three steps: (1) the set of absolute differences, $\{d_i\}$, between $\{w_i\}$ and their median value is evaluated; (2) the median value of $\{d_i\}$ is computed, namely $M(\{d_i\})$; (3) the values $w_i$ in the original set for which $d_i$ is higher than three times $M(\{d_i\})$ are replaced by the median of $\{w_i\}$. The mean of the resulting filtered set has been considered to be representative of each bin.

## Results and discussion

**Figure 1:**
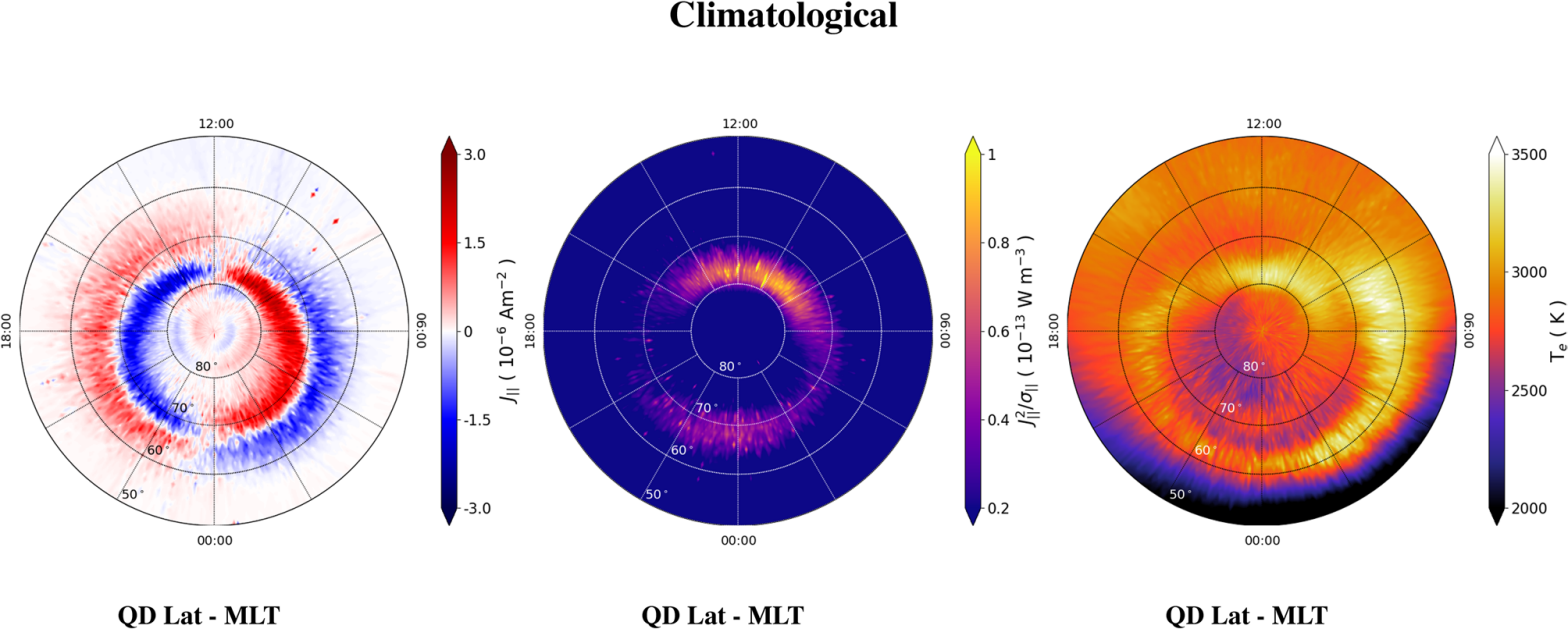
Maps in QD latitude vs MLT coordinates of FAC density, $J_{||}$, (left panel); dissipated power density, $J_{||}^2/\sigma _{||}$, (central panel); and electron temperature, $T_e$, (right panel) in the Northern hemisphere obtained by considering 6 years of Swarm A data. The values of $J_{||}$ are saturated below -3$\times 10^{-6}$ A m$^{-2}$ and above 3$\times 10^{-6}$ A m$^{-2}$; the values of $J_{||}^2/\sigma _{||}$ are saturated below 0.2$\times 10^{-13}$ W m$^{-3}$ and above 1.0$\times 10^{-13}$ W m$^{-3}$; the values of $T_e$ are saturated below 2000 K and above 3500 K.

### Climatological behavior of the dissipated power density

Figure 1 shows climatological (i.e., obtained by considering six years of Swarm A data) maps of $J_{||}$ (left panel), $J_{||}^2/\sigma _{||}$ (central panel) and $T_e$ (right panel) in the Northern hemisphere. The bulk of $J_{||}$ values distribution ranges between $\simeq$ − 3.0 $\times$ 10$^{-6}$ A m$^{-2}$ and $\simeq$ 3.0 $\times$ 10$^{-6}$ A m$^{-2}$, consistent with early statistical studies. Concerning $J_{||}$, negative values correspond to currents flowing away from the ionosphere, while positive ones correspond to currents flowing into the ionosphere. These patterns are consistent with those shown, e.g., in the milestone work by Iijima and Potemra^30^. In particular, the fast-cadence measurements acquired during 6 years by Swarm A allow identifying features such as regions R1 and R2^30^, and features at even higher latitudes, such as region R0^31^. Particularly interesting are the $J_{||}$ features taking place in the Northern hemisphere’s polar cap. Their patterns share the same sign as the currents in region R2, and are directed towards the ionosphere in the dusk sector and from the ionosphere in the dawn sector. This pattern is consistent with the results found by Eriksson et al.^32^, who analyzed data acquired by the FAST satellite^33^ flying along dawn-dusk orbits. The authors reported evidence for a system of six FACs rather than the usual system of four FACs associated with regions R1 and R2. They also argued that the position of these patterns is strongly correlated with the polarity of the East-West component of the interplanetary magnetic field.

The power density dissipated, $J_{||}^2/\sigma _{||}$, ranges between 0 and 1.47$\times 10^{-14}$ W m$^{-3}$. We estimated the statistical error for $J_{||}^2/\sigma _{||}$ by using a bootstrap method that consisted in the following steps: (i) for each bin, we selected 1000 different subsets sized at 60% of the total number of values falling within the bin; (ii) the median value of each subset was computed; (iii) the standard error of the subsets was computed and assumed as the uncertainty associated with $J_{||}^2/\sigma _{||}$. Following this procedure, which was also used by Giannattasio et al.^8,19^ to estimate the uncertainties for $\sigma _{||}$, we found a maximum error of $\simeq$ 2.5%. The central panel of Fig. 1 shows that the dissipated power density occurs in a thin range of QD latitudes between 70$^\circ$ and 80$^\circ$ in the dayside and between 65$^\circ$ and 70$^\circ$ in the nightside. The highest values of $J_{||}^2/\sigma _{||}$ occur mainly at high QD latitudes in the dayside due to the increase in current density therein. In particular, $J_{||}^2/\sigma _{||}$ increases above all between 08:00 and 16:00 MLT, in correspondence with the cusp region^31^, where intense particle precipitation from the dayside open magnetosphere occurs. In the nightside, enhancements of $J_{||}^2/\sigma _{||}$ are observed between 65$^\circ$ and 70$^\circ$ of QD latitude at all MLTs from dusk to dawn. This is not surprising as also this region was recognized to be characterized by intense particle precipitation from the nightside magnetosphere^34^.

$T_e$ ranges between 1583 K and 3702 K. The application of the bootstrap method described above provided a maximum uncertainty of $\simeq$ 0.4%. $T_e$ maps are fully consistent with those derived by Pignalberi et al.^35^ from 7 years of Swarm data acquired at 1 s cadence. The highest values of $T_e$ occur at QD latitudes between 60$^\circ$ and 70$^\circ$ and 06:00 and 08:00 MLT, and at QD latitudes between 75$^\circ$ and 80$^\circ$ and between 08:00 and 12:00 MLT. However, the $T_e$ enhancement at those latitudes extends between 06:00 and 14:00 MLT in correspondence with region R0 and the magnetic cusp^31^ and is due to intense particle precipitation in response to the magnetosphere-ionosphere coupling in the dayside^36^. As a general feature, $T_e$ in the dayside is higher than in the nightside, especially at QD latitudes $\lesssim$ 60$^\circ$, due to solar illumination and the contribution of EUV ionization between 06:00 and 18:00 MLT. On the other hand, an increase in $T_e$ in the nightside at QD latitudes $\sim$ 60$^\circ$ and $\sim$ 70$^\circ$ is present. The rise $T_e$ at $\sim$ 60$^\circ$ is probably due to the joint action of particle precipitation from the nightside magnetosphere (from both the magnetotail and the plasmasphere, where electrons heated by collisions with ions diffuse along the geomagnetic field lines) and the decreased collisional cooling at the edge of the electron depletion region at middle latitudes^37^. The drop in $T_e$ at QD latitudes around 65$^\circ$ and 75$^\circ$ may be in correspondence with the amplification of horizontal Hall and Pedersen currents due to the coupling between the magnetotail and the nightside ionosphere. These currents are known to flow and dissipate (with a consequent increase in $T_e$) at 90–110 km altitude, i.e., far below the Swarm A altitudes considered in this work.

It is interesting to notice that while in the dayside, at QD latitudes $\sim$ 80$^\circ$ and MLTs between 08:00 and 14:00, enhancements of $T_e$ are co-located with an increase in $J_{||}^2/\sigma _{||}$, in the nightside, at QD latitudes around $\sim$ 65$^\circ$ and MLTs between 20:00 and 04:00, a drop in $T_e$ is co-located with an increase in $J_{||}^2/\sigma _{||}$. Vice versa, at QD latitudes around $\sim$ 60$^\circ$ and $\sim$ 70$^\circ$, a drop in $J_{||}^2/\sigma _{||}$ is co-located with an increase in $T_e$ from dusk to 08:00 MLT. In other words, increases in $T_e$ are associated with increases in $J_{||}^2/\sigma _{||}$ in the dayside at high QD latitudes (in the cusp region), while increases in $T_e$ are associated with decreases in $J_{||}^2/\sigma _{||}$ and vice versa in the nightside at QD latitudes between 60$^\circ$ and $\sim$ 70$^\circ$ (in the auroral and subauroral regions). We speculate on this behavior by considering that parallel electrical conductivity strongly depends on $T_e$ (Eq. 5). In the dayside, increased dissipation takes place where $J_{||}^2$ substantially exceeds $\sigma _{||}$. Thus, despite of high values of $T_e$, and consequently of $\sigma _{||}$, $J_{||}$ is so high that an overall high dissipation occurs. This might be consequence of two facts: (1) $T_e$ increases in response to dissipation; (2) precipitating electrons are suprathermal, i.e. their bulk velocity is higher than their thermal velocity^38^; in other words, their energy, which is proportional to $J_{||}^2$, does not scale as $\propto kT_e$, as it is the case for electrons accelerated by waves^39^, by parallel electric fields, or by ion-acoustic turbulence^40^, just to mention a few mechanisms. In the nightside, increased dissipation occurs where field-aligned currents increase and $T_e$ (and, thus, $\sigma _{||}$) is low, i.e., at $\sim$ 65$^\circ$ of QD latitude. Above and below this latitude, i.e., at $\sim$ 70$^\circ$ and $\sim$ 60$^\circ$ of QD latitude, dissipation decreases as $J_{||}$ decreases and/or $T_e$ increases.

### Variation of dissipated power density with geomagnetic activity

**Figure 2:**
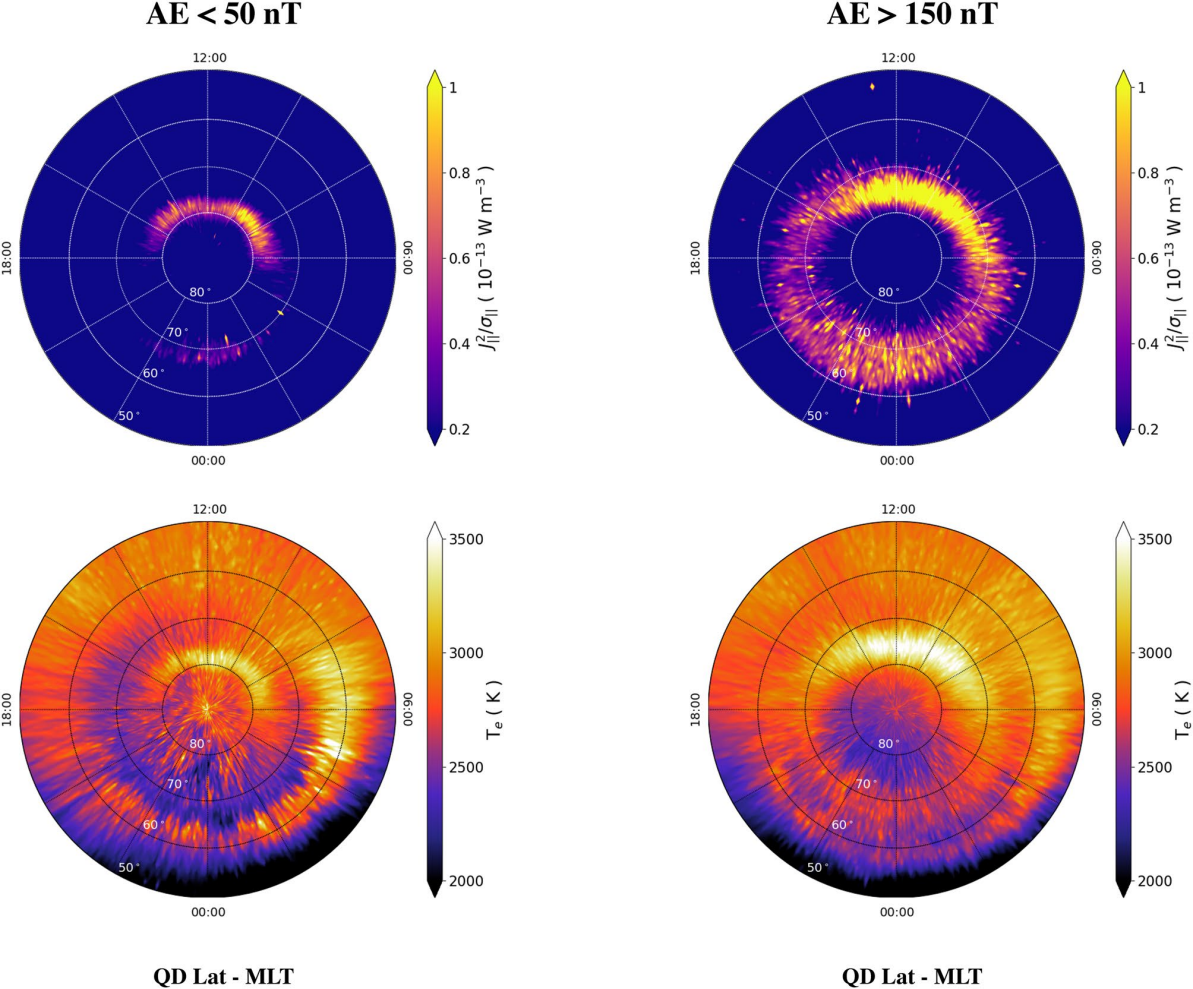
Maps in QD latitude vs MLT coordinates of dissipated power density, $J_{||}^2/\sigma _{||}$ (top row), and electron temperature, $T_e$ (bottom row) in the Northern hemisphere during quiet (left column) and disturbed (right column) conditions. The values of $J_{||}^2/\sigma _{||}$ are saturated below 0.2 $\times 10^{-13}$ W m$^{-3}$ and above 1.0 $\times 10^{-13}$ W m$^{-3}$. The values of $T_e$ are saturated below 2000 K and above 3500 K.

**Figure 3:**
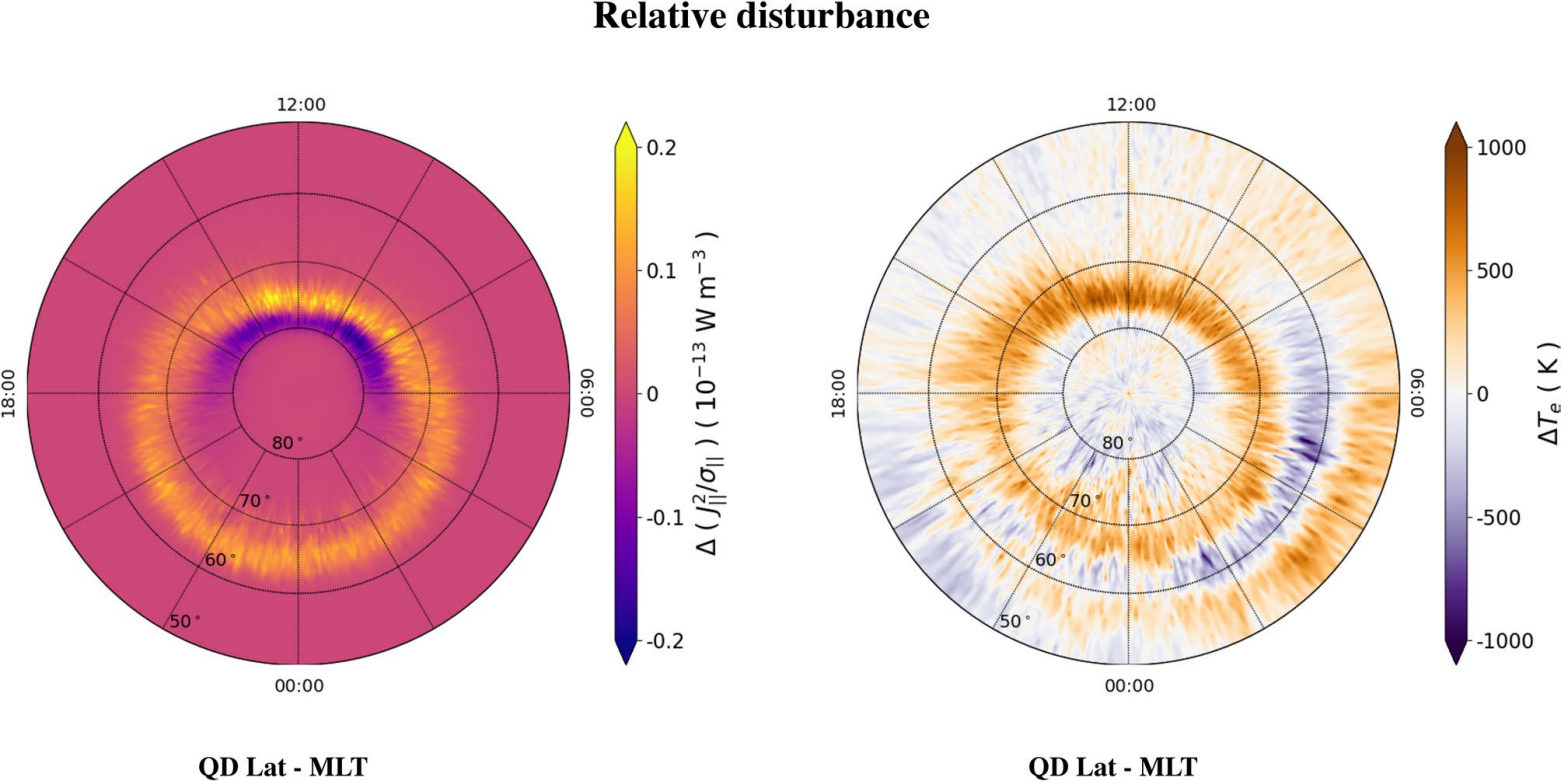
Difference maps in QD latitude vs MLT coordinates of dissipated power density, $J_{||}^2/\sigma _{||}$, (left panel) and electron temperature, $T_e$, (right panel) in the Northern hemisphere. Difference maps are retrieved by subtracting maps obtained during quiet conditions (AE < 50 nT) from maps obtained during disturbed conditions (AE > 150 nT). The values of $\Delta (J_{||}^2/\sigma _{||})$ are saturated below -0.2 $\times 10^{-13}$W m$^{-3}$ and above 0.2 $\times 10^{-13}$ W m$^{-3}$; the values of $\Delta T_e$ are saturated below − 1000 K and above 1000 K.

In order to study the dependence of dissipated power density on the geomagnetic activity, we investigated two different levels of geomagnetic activity, as measured by 1-min Auroral Electrojet index (AE) as a proxy^41^, namely AE < 50 nT (quietness) and AE > 150 nT (disturbance). The AE index, which is computed starting from variations in the horizontal component of the geomagnetic field at 12 observatories in the auroral region of the Northern hemisphere, is provided by the World Data Center for Geomagnetism in Kyoto (http://wdc.kugi.kyoto-u.ac.jp/aedir/) up to 28 February, 2018. Thus, AE index time series considered in this analysis ranges between 1 April 2014 and 28 February 2018. Despite this time series is 22 months shorter than the other series used in this work (namely, $T_e$, $n_e$ and $J_{||}$) we decided to use it for studying processes occurring at QD latitudes $\gtrsim$ 50$^\circ$ mainly for two reasons: (1) AE index is still the official standard recognized by the International Association of Geomagnetism and Aeronomy; (2) AE index is by far the most used index worldwide, and the thresholds used in this work to select different geomagnetic activity levels can be easily compared with those found in the literature.

The top row of Fig. 2 shows maps of $J_{||}^2/\sigma _{||}$ in the Northern hemisphere during quiet (AE < 50 nT, on the left) and disturbed (AE > 150 nT, on the right) geomagnetic conditions, respectively. The maximum value of $J_{||}^2/\sigma _{||}$ is $7.85\times 10^{-13}$ W m$^{-3}$ with a maximum error computed with the bootstrap method of 5.81% during quiet conditions and $5.21\times 10^{-13}$ W m$^{-3}$ with a maximum error of 1.00% during disturbed conditions, respectively. This means that the absolute maximum value of $J_{||}^2/\sigma _{||}$ occurs during quiet conditions, when dissipation is intense only in the cusp region, while it is faint and confined in a very narrow range of QD latitudes $\sim$ 70$^\circ$ in the nightside due to the reduced magnetosphere–ionosphere dynamics. Thus, during quiet conditions only the cusp dynamics is able to deposit conspicuous amounts of energy in the ionosphere. The smallest dissipation is observed after dusk and before dawn. During disturbed conditions the patterns of dissipation change substantially. In this case, dissipation occurs at all MLTs in a broader range of QD latitudes with respect to the quiet case, up to 10$^\circ$ wide in the nightside, expanding to lower latitudes down to $\sim$ 62$^\circ$ between 22:00 and 00:00 MLT.

We studied the behavior of $T_e$ under the same geomagnetic conditions (bottom row of Fig. 2). $T_e$ ranges between 1191 and 2363 K with a maximum error of 0.1% computed with the bootstrap method during quiet conditions, while it ranges between 1487 and 3782 K with a maximum error of 1% during disturbed conditions. In both cases the highest values occur in the cusp region, at noon during quiet conditions and between 08:00 and 12:00 MLT during disturbed conditions, where intense particle precipitation occurs^42^. A local maximum is also observed during quiet conditions at $\sim$ 60$^\circ$ of latitude in the predawn sector. As expected, also the enhancement of $T_e$ expands to lower latitudes with the increase of geomagnetic activity, e.g. from $\sim$ 80$^\circ$ to $\sim$ 75$^\circ$, in the dayside. In the nightside, the features observed in the climatological case (see Fig. 1) at $\sim$ 60$^\circ$ and $\sim$ 70$^\circ$ QD latitude appear at slightly lower latitudes (at $\sim$ 57$^\circ$ and $\sim$ 67$^\circ$, respectively) passing from quiet to disturbed conditions. In accordance, the equatorward drop in $T_e$ reduces its latitudinal extension and moves equatorward from $\sim$ 65$^\circ$ to $\sim$ 60$^\circ$. Regarding the link between $J_{||}^2/\sigma _{||}$ and $T_e$, we notice that: (1) in the cusp regions $J_{||}^2/\sigma _{||}$ enhancements are always co-located with $T_e$ ones; (2) in the nightside and for quiet conditions a drop in $T_e$ is co-located with an enhancement of $J_{||}^2/\sigma _{||}$, while for disturbed conditions $T_e$ and $J_{||}^2/\sigma _{||}$ features are quite co-located. A possible explanation for this behavior could be the following. The cusp region is a site of permanent particle precipitation, whose energy release allows increasing $T_e$ without decreasing $J_{||}^2/\sigma _{||}$ itself. In the nightside and for quiet conditions, power density dissipation increases due to the decrease of $T_e$. The resulting dissipation should result in increased $T_e$, but the presence of energy fluxes towards the surrounding colder regions (via, for instance, zonal horizontal winds) could trigger a mechanism that keeps the physical conditions of the ionosphere stationary on the nightside at middle and high latitudes. During disturbed conditions, particle precipitation also dominates the nightside dynamics at the same latitudes, probably due to the joint effect of both the increased energy of particle precipitation and the decreased $n_e$ with the following decrease of collisional cooling. In this case, the sites of dissipation are co-located with those of increased $T_e$, likewise in the cusp regions.

The variation of $J_{||}^2/\sigma _{||}$ and $T_e$ with increasing geomagnetic activity is better pointed out by mapping the difference between such quantities during disturbed and quiet conditions. The results are shown in Fig. 3, where the mentioned difference is indicated by $\Delta$. For example, in the case of $T_e$, we define $\Delta T_e\equiv T_{e,AE > 150 nT}-T_{e, AE < 50 nT}$. The same definition applies to $\Delta (J_{||}^2/\sigma _{||})$. Figure 3 suggests that FACs mainly dissipate in the auroral regions. During quiet conditions, dissipation occurs mainly in the dayside, in correspondence of the cusp (negative values, in blue), while during disturbed conditions dissipation occurs along the whole auroral regions at slightly lower QD latitudes (positive values, in yellow). This is consistent with the expansion of field-aligned current patterns and auroral regions to lower latitudes during disturbed conditions^30,43,44^. An important feature of relative disturbance is that, in correspondence of dissipation enhancements, $T_e$ increases accordingly. In fact, passing from quiet to disturbed conditions positive values of $\Delta (J_{||}^2/\sigma _{||})$ correspond to positive values of $\Delta T_e$. On the other hand, passing from disturbed to quiet conditions negative values of $\Delta (J_{||}^2/\sigma _{||})$ correspond to negative values of $\Delta T_e$. The nightside during quiet conditions represents an exception, since between 22:00 and 02:00 MLT and between 70$^\circ$ and 80$^\circ$ of QD latitude there is a slight decrease in $J_{||}^2/\sigma _{||}$ accompanied by a slight increase in $T_e$. This enhancement in $\Delta T_e$ is probably due to the drop of $T_e$ in the same region in quiet conditions, which could be associated with a polar hole^36^, i.e., a region of decayed $n_e$ due to both the lack of photoionization and a slow convection pattern.

## Summary and conclusions

In the context of magnetosphere-ionosphere coupling, the characterization of FACs plays a key role. One important question to answer is: How is energy deposited in the ionosphere by FACs? Dissipation of FACs may be the answer in the topside ionosphere. For the first time, we showed the patterns of dissipation of FACs and their variation with geomagnetic activity at Swarm altitudes, and pointed out that during disturbed conditions they are associated with increasing values of $T_e$. This suggests that dissipation of FACs may be responsible for the increased energy, and thus $T_e$, of plasma in the topside ionosphere. In particular, the main results of this work concerning $J_{||}^2/\sigma _{||}$ can be summarized as follows:

- Features associated with power density dissipation occur in a thin range of QD latitudes in the cusp region and between 65$^\circ$ and 70$^\circ$ in the nightside;
- In correspondence with higher AE index values dissipation features intensify and expand towards lower latitudes. Moreover, while during quiet conditions dissipation occurs mainly in the cusp region, during disturbed conditions it takes place in the auroral region at all MLTs;
- In both the cusp and auroral/subauroral regions of the disturbed nightside, features of enhanced $J_{||}^2/\sigma _{||}$ are co-located with features of increased $T_e$ probably due to both the suprathermal nature of precipitating electrons and the increase of $T_e$ after dissipation. On the contrary, in the quiet nightside, when the contribution of suprathermal incoming electrons is small, $J_{||}^2/\sigma _{||}$ slightly increases with decreasing $T_e$ (i.e., with decreasing $\sigma _{||}$).

Our results support the evidence that parallel dissipation is de-facto not zero at Swarm altitudes, even though its strength is several orders of magnitude lower, with respect to dissipation due to perpendicular currents that occur at much lower altitudes. Moreover, this result is obtained by neglecting the fine structure of FACs, as it is based on large-scale measurements, so that nothing can be said on the local dissipation at scales finer than $\sim$ 1 km. Indeed, if assuming a mainly filamentary FACs’ structure^45–47^, observed dissipation is very likely to be underestimated. This is a point that needs to be investigated in future work. A correct evaluation of the parallel electric field in the regions where we observe a significant enhancement of parallel dissipation during disturbed periods requires the possibility to use direct in situ measurements of the electric field. This point will be investigated in a subsequent work using, for instance, data from the Chinese mission “CSES-01”. This study will be extended also to the analysis of power density dissipation in the Southern hemisphere. An overriding objective to be pursued in the future is to compute the correlation and model the complex link between electron temperature variations and FACs dissipation, also considering temperature variations due to other physical mechanisms, such as, for instance, particle precipitation. This should imply the application of plasma energy balance equations by evaluating plasma pressure, plasma velocity, energy flux, among the others, together with flux and temperature gradients in a three-dimensional geometry. Another point that needs to be thoroughly investigated in the future is the height-integrated FAC dissipation, which might be relevant considering the extent of FACs coupling magnetosphere and ionosphere. However, a rigorous estimation of this quantity is by no means trivial and, to correctly evaluate the parallel electrical conductivity, would imply the introduction of models of vertical profiles of collision rate, density and temperature of all species involved. In the future, it would be also interesting to study the features of dissipation with the solar activity and the different magnetosphere topological configurations as described, e.g., by the different components of the interplanetary magnetic field carried on by the solar wind. The seasonal dependence also should be deepened, as the different sunlit conditions crucially affect the Earth’s environment. Finally, it would be interesting to estimate the contribution of dissipation due to diamagnetic currents, for instance in relation with the occurrence of irregularities in the auroral oval^48^.

## Data Availability

Swarm data can be accessed at https://swarm-diss.eo.esa.int. Plasma data used in this work can be found at: https://swarm-diss.eo.esa.int/#swarm%2FLevel1b%2FLatest_baselines%2FEFIxLPI%2FSat_A. FAC data used in this work can be found at: https://swarm-diss.eo.esa.int/#swarm%2FLevel2daily%2FLatest_baselines%2FFAC%2FTMS%2FSat_A.
